# Tailoring Catalysts for CO_2_ Hydrogenation: Synthesis and Characterization of NH_2_–MIL–125 Frameworks

**DOI:** 10.3390/molecules30071458

**Published:** 2025-03-25

**Authors:** Leidy Figueroa-Quintero, Tomás Cordero-Lanzac, Enrique V. Ramos-Fernandez, Unni Olsbye, Javier Narciso

**Affiliations:** 1 Inorganic Chemistry Department, Laboratory of Advanced Materials, University Materials Institute of Alicante, University of Alicante, 03080 Alicante, Spain; leidy.figueroa11@ua.es (L.F.-Q.); enrique.ramos@ua.es (E.V.R.-F.); 2 SMN Centre for Materials Science and Nanotechnology, Department of Chemistry, University of Oslo, Sem Saelands Vei 26, 0371 Oslo, Norway; t.c.lanzac@smn.uio.no (T.C.-L.); unni.olsbye@kjemi.uio.no (U.O.)

**Keywords:** MOF, CO_2_ hydrogenation, post-synthetic modification

## Abstract

Copper nanoparticles have been integrated onto the framework of modified NH_2_–MIL–125(Ti), a metal–organic framework (MOF), and evaluated as catalysts for converting CO_2_ into valuable products. The modified MOF was achieved through a post-synthetic modification process involving the partial replacement of titanium with zirconium or cerium within the MOF’s structure. The objective behind this alteration is to create a synergistic effect between the MOF, serving as a support matrix, and the embedded copper nanoparticles, thereby enhancing the performance of the catalyst. The obtained catalysts were characterized and evaluated in the hydrogenation of CO_2_ to methanol under different experimental conditions, reaching CO_2_ conversions of up to 5%, with a selectivity towards methanol that reached values of up to 60%. According to the obtained results, the catalyst composed of Ti, Zr and Cu stood out for having the highest CO_2_ conversion and selectivity towards methanol, in addition to practically inhibiting the production of methane. These results demonstrate that the interaction of the framework with the Cu nanoparticles, and thus its catalytic properties, can be changed by modifying the properties of the MOF.

## 1. Introduction

The efficient conversion of carbon dioxide (CO_2_) into high-value products through various processes represents a highly promising strategy for mitigating greenhouse gas emissions. Among the products resulting from CO_2_ reduction, methanol stands out as a particularly significant compound, given the growing global demand that exceeds 250 million tons per year. Among methanol direct applications, it is used as liquid fuel that is easy to store and transport, thus emerging as a promising alternative to conventional fossil fuels [1]. In addition to its application as a fuel, methanol serves as a precursor for a variety of essential chemical processes in organic synthesis, pesticide or drug production or the coatings and automotive industries, among others [2,3,4].

In this context, obtaining methanol from CO_2_ hydrogenation is a promising alternative in the search for CO_2_ revalorization. This reaction is exothermic (1) and competes with the reverse water–gas shift reaction (2). In addition, it is feasible to carry out the hydrogenation of carbon monoxide (CO) to obtain methanol, as represented (3).

(1) $${\mathrm{CO}}_{2}+{3H}_{2}↔{\mathrm{CH}}_{3}\mathrm{OH}+H_{2}O;\;\;\;\;{∆G}_{298K}^{0}=\;–49.5\;\mathrm{KJ}/\mathrm{mol}$$

(2) $${\mathrm{CO}}_{2}+H_{2}↔\mathrm{CO}+H_{2}O;\;\;\;\;{∆G}_{298K}^{0}=\;+41.2\;\mathrm{KJ}/\mathrm{mol}$$

(3) $$\mathrm{CO}+{2H}_{2}↔{\mathrm{CH}}_{3}\mathrm{OH};\;\;\;\;{\;∆G}_{298K}^{0}=\;–90.6\;\mathrm{KJ}/\mathrm{mol}$$

(4) $${\mathrm{CO}}_{2}+{4H}_{2}↔{\mathrm{CH}}_{4}+{2H}_{2}O;\;\;\;\;{∆G}_{298K}^{0}=\;–164.6\;\mathrm{KJ}/\mathrm{mol}$$

This reaction exhibits a high-temperature-constrained equilibrium, which underlines the need to find an efficient catalyst that can operate at the desired low temperature [5]. This is why numerous researchers have directed their efforts towards the design of a catalytic system that facilitates CO_2_ conversion, being thermally stable and at the same time selective towards methanol production. Taking into account these characteristics, catalysts based on transition metals (mostly Cu [6]) and other metals such as in [7,8,9] and Pd [10,11], among others, have been developed over the years [8,11,12,13,14,15,16,17].

From this perspective, metal–organic frameworks (MOFs) have emerged as key players in the field of catalysis and gas storage. These porous crystalline structures, composed of metal clusters and organic linkers [18,19], possess unique properties that have allowed their application in a wide variety of catalytic processes. In particular, post-synthetic modification of their metal nodes has proven to be an effective strategy to improve their stability and catalytic activity [20]. The incorporation of metals such as Zr and Ce can alter the electronic properties of the catalytic centers, optimizing the conversion of CO_2_ [20,21,22]. Moreover, MOFs stand out for their ability to modify their secondary building units (SBUs) by post-synthetic strategies, which has boosted their study in heterogeneous catalysis [23,24,25,26,27].

Several MOFs have been studied in CO_2_ hydrogenation, revealing promising results [28,29]. Bing An et al. [30] studied the confinement of Cu/ZnO_x_ nanoparticles in MOFs as catalysts for this reaction. The synthesized catalysts showed very high activity with a space time yield of up to 2.59 g_MeOH_ kgCu^−1^h^−1^, 100% selectivity to methanol and high stability for 100 h on stream. In a similar approach, Bunyarat Rungtaweevoranit et al. [6] demonstrated how the activity of a copper catalyst could be promoted by a Zr-based MOF (UiO–66). These researchers observed that the performance of the synthesized catalyst exceeded that of the reference catalyst (Cu/ZnO/Al_2_O_3_), showing a constant 8-fold higher yield and 100% selectivity to methanol. In most of these studies, the MOF structure has been identified as a modifier of the electronic configuration of Cu nanoparticles, leading to the stabilization of the Cu nanoparticle and conferring unique properties. Velisoju et al. [31] discovered that the strategic anchoring of Cu nanoparticles onto the ZIF-8 framework results not only in a very selective catalyst but also showed remarkable methanol productivity. Impressively, this catalyst surpasses the performance of current commercial options and all other MOF-based catalysts for this specific reaction. This finding underscores the exceptional characteristics of MOFs that render them particularly effective for methanol synthesis.

Previous research has focused on the interaction of Cu with structures composed of Zr [6,31,32], Ce [21,33,34] or Zn [21]. Interestingly, MOFs constructed with Ti have not been explored for this reaction, despite their potential to exhibit distinctive properties, combining their thermal and chemical stability with high porosity and catalytic properties. In this regard, within the MOF family, NH_2_–MIL–125 has captured the attention of the scientific community. Its crystalline structure, formed by Ti octahedra connected by organic ligands (amino terephthalic acid), gives it a large surface area and a marked porosity. These characteristics make this material promising for gas storage and catalysis applications. The amino (–NH_2_) functionalities present in the structure contribute to its catalytic properties, making NMIL–125 particularly attractive for specific reactions such as liquid phase oxidative desulfurization [35], photocatalytic degradation of pollutants [36], photocatalytic CO_2_ reduction [37] or CO_2_ cycloaddition to propylene oxide [38], among others.

In this study, we carried out a post-synthetic modification of NH_2_–MIL–125 by partial metal exchange with elements such as cerium (Ce) and zirconium (Zr). The introduction of these elements not only strengthened the stability of NH_2_–MIL–125 but also evidenced improvements in its catalytic performance. This modified NH_2_–MIL–125 (M: Ce or Zr) was used as a support for copper nanoparticles. We have found that the Zr- and Ce-modified catalysts have different properties to the unmodified material, especially the Zr one, showing better yield and selectivity to methanol.

## 2. Results and Discussion

### 2.1. Characterization of Catalysts

**Figure 1:**
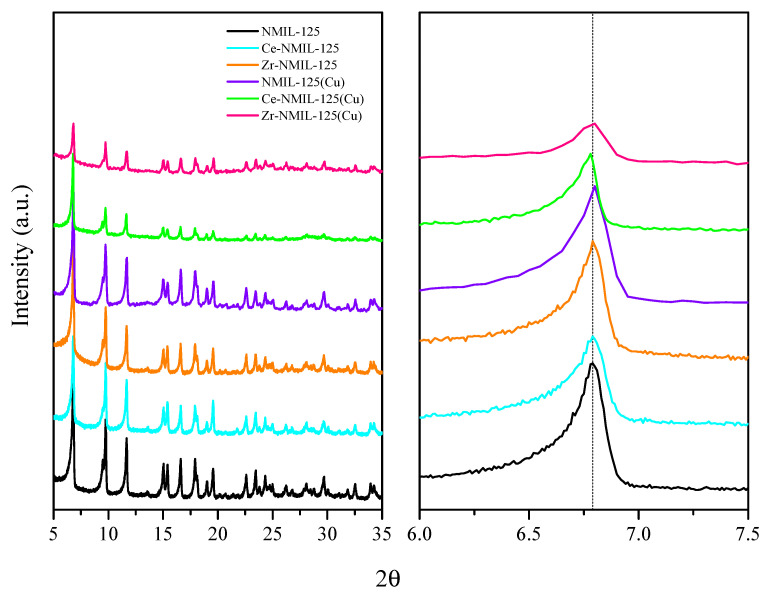
XRD patterns of the as-synthesized NMIL–125 and the mixed metal NMIL–125-based catalysts.

The crystallinity of the synthesized catalysts was evaluated by powder X-ray diffraction, as presented in Figure 1. The pattern of NMIL–125 shows characteristic peaks consistent with the known structure, aligning with the results of previous studies [39,40]. Upon partial metal exchange, the crystallinity of the MOF is not compromised, only a slight shift of the main diffraction peak towards lower 2 theta values is observed for the modified samples. As expected for the M–NMIL–125 materials, the unit cell expanded with respect to that of the pristine MOF, due to the replacement of Ti^4+^ atoms by Ce^4+^ or Zr^4+^ atoms, whose atomic radiuses are larger than those of Ti^4+^ (Ti^4+^: 0.68 Å, Ce^4+^: 1.01 Å, Zr^4+^: 0.80 Å), corroborating the successful incorporation of the metals into the SBU of the MOF. Unit cell expansion resulted in the following lattice parameters: a = 18.695 Å, b = 18.695 Å, c = 18.110 Å for both Ce–NMIL–125 and Zr–NMIL–125, with the following parameters corresponding to NMIL–125 without post-synthetic modification: a = 18.654 Å, b = 18.654 Å, c = 18.174 Å.

The metal content measured in each of the materials by Inductively Coupled Plasma–Optical Emission Spectroscopy (ICP–OES) corroborates the successful incorporation of the metals in the starting MOF, as shown in the table below (Table 1). The results indicate that the second metal, whether Zr or Ce, is incorporated in very similar amounts. Additionally, the final loadings of Cu range from 27.4 to 29.5 at% in all cases, making the materials comparable. To better understand the incorporation of metals at the molecular level, the metal contents were also expressed as formula units, as detailed in Table 1. Formula unit analysis revealed that metal exchange with Ti did not result in significant changes in the SBU, as reflected by the minimal variation in unit cell parameters (less than 1%) for the modified samples compared to the original NMIL–125. These findings highlight the stability of the SBU during metal exchange and further corroborate the successful and consistent incorporation of metals into the MOF structure.

**Figure 2:**
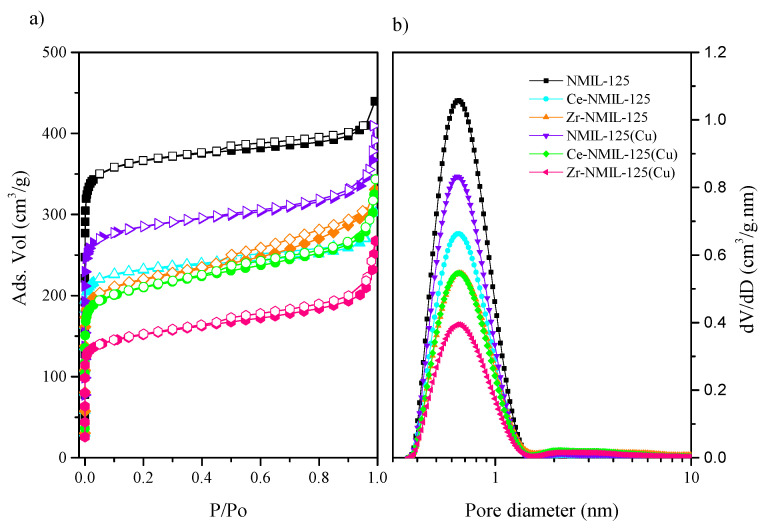
(**a**) N_2_ adsorption isotherms, (**b**) pore size distribution of the as-synthesized catalysts.

The textural properties of the catalysts were also evaluated, performed by means of N_2_ adsorption isotherms at 196 °C, and are presented in Figure 2a. According to the IUPAC classification, the isotherms obtained correspond to type I [41]. For all catalysts, a significant increase in the amount of nitrogen adsorbed was observed at low relative pressures, reaching a plateau after a relative pressure of 0.2. This behavior is characteristic of microporous materials, further corroborated by the pore size distribution (Figure 2b). Although all catalysts show this behavior, it is evident that the introduction of new metals causes a small decrease in the surface area of the bimetallic catalysts (NMIL–125(Cu), Ce-NMIL–125 and Zr-NMIL–125). Furthermore, the incorporation of Cu nanoparticles in the bimetallic catalysts (Ce–NMIL–125(Cu) and Zr–NMIL–125(Cu)) generates a more attenuated decrease in their textural properties, observing a decrease in the specific surface area with respect to the starting MOF from 1114 m^2^/g to 659 m^2^/g and 455 m^2^/g, respectively (see Table 2). That said, it is important to note that the surface area of these catalysts is very large compared to other catalysts used for this reaction.

**Figure 3:**
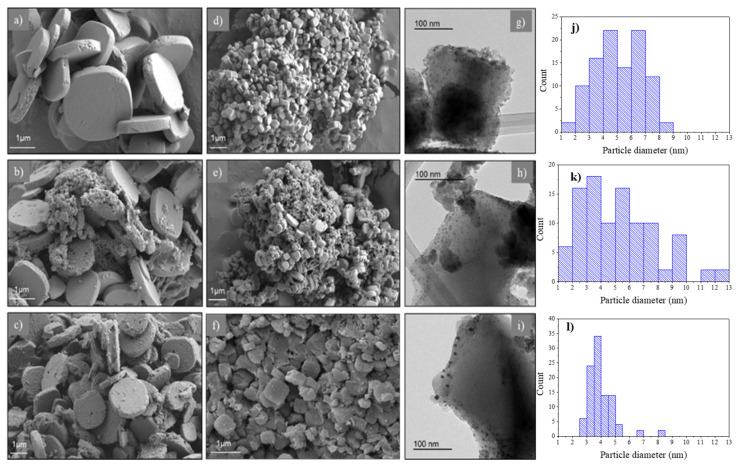
FESEM images of: (**a**) NMIL–125, (**b**) Ce–NMIL–125, (**c**) Zr–NMIL–125, (**d**) NMIL–125(Cu), (**e**) Ce–NMIL–125(Cu), (**f**) Zr–NMIL–125(Cu). TEM images of: (**g**) NMIL–125(Cu), (**h**) Ce–NMIL–125(Cu) and (**i**) Zr–NMIL–125(Cu). And particle size distribution of: (**j**) NMIL–125(Cu), (**k**) Ce–NMIL–125(Cu) and (**l**) Zr–NMIL–125(Cu).

The morphology of the catalysts was carefully examined using field emission scanning electron microscopy (FESEM) and transmission electron microscopy (TEM). The images captured are depicted in Figure 3. Figure 3a illustrates the starting MOF (NMIL–125), which exhibits uniform disk-shaped particles, with particle size ranging from 2 to 3 µm, agreeing with the findings of other researchers [35]. Figure 3b,c show the Ce–NMIL–125 and Zr–NMIL–125 catalysts, respectively. The formation of agglomerates around the disks can be seen. And finally, the influence of the inclusion of the copper nanoparticles on the catalysts can be observed in Figure 3d–f, which causes a remarkable decrease in the particle size (0.5 µm) and suggests a possible recrystallization in the structure of the materials. On the other hand, TEM images (Figure 3g–i) show the successful formation of these nanoparticles (Cu), which are uniformly dispersed in the matrix of the catalysts. The diameter of the copper nanoparticles was mainly in the range of 4–7 nm (average distribution of 5.14 nm) for NML-125(Cu), 3–4 nm (average distribution of 3.96 nm) for Ce-NMIL–125(Cu) and 2–6 nm (average distribution of 5.23 nm) for Zr-NMIL–125(Cu). These values were obtained from measurements of 100 randomly selected particles (Figure 3j–l).

**Figure 4:**
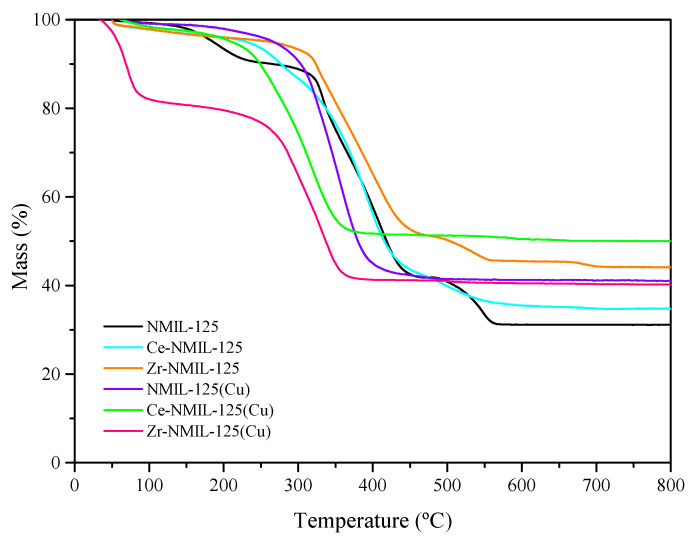
TGA traces measured in air of NMIL–125, Ce–NMIL–125, Zr–NMIL–125, NMIL–125(Cu), Ce–NMIL–125(Cu), Zr–NMIL–125(Cu).

The thermal stability of the catalysts was evaluated by thermogravimetric analysis from 25 °C to 800 °C under air atmosphere (Figure 4). The TG curves corresponding to NMIL–125 and Zr–NMIL–125(Cu) are consistent with published TG data for this MOF in other works [41,42] and reveal a weight loss mainly in two steps. The first weight loss between 80 and 200 °C corresponds to the outflow of host molecules adsorbed in the pores, including residual solvent molecules. In the case of Zr-NMIL–125(Cu), a more pronounced initial mass loss was observed in this temperature range, suggesting a higher retention of residual solvent molecules, particularly methanol, used during synthesis and post-synthetic treatments. This phenomenon has been reported in previous studies, where it has been observed that Zr incorporation affects the thermal stability and adsorption capacity of polar molecules [43]. The main weight loss, between 250 and 600 °C, corresponds to a series of degradation processes of the MOF structure and the decomposition of the aminoterephthalic acid of the structure [44,45], finally producing an amorphous TiO_2_ residue [40] for the starting NMIL–125 parent MOF. As for the NMIL–125(Cu), Zr–NMIL–125, Ce–NMIL–125 and Ce–NMIL–125(Cu) catalysts, the weight loss occurs in a single step, starting at 200 °C, and corresponds to the decomposition of the aminoterephthalic acid and collapse of the structure. At this point, it is important to highlight the thermal stability exhibited by Zr–NMIL–125, which exhibits a weight loss at a higher temperature than the other catalysts, even at a temperature slightly higher than that of the starting MOF. This suggests that the combination of NMIL–125 with Zr can modestly improve its thermal stability, which is consistent with the well-known stable coordination bond of Zr terephthalic that forms robust MOFs [46]. On the other hand, the presence of well-dispersed Cu nanoparticles facilitates the thermal decomposition of the MOF structure at a high temperature [29], since copper is a good catalyst for oxidation.

**Figure 5:**
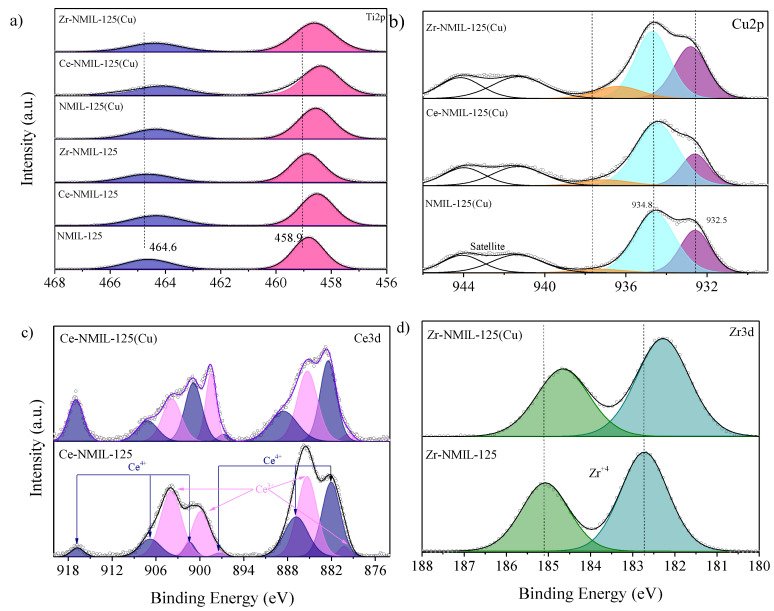
XPS spectra for the catalysts: (**a**) Ti2p, (**b**) Cu2p, (**c**) Ce3d, (**d**) Zr3d.

XPS analyses were performed to obtain more information about the composition and the chemical and electronic states of each of the elements of the catalysts. The surface elemental composition of the catalysts is shown in Table S1. The spectra represented in Figure 5 and Figure S1 confirm the presence of Ti, C, N and O species plus Cu, Zr and Ce in those post-treated MOFs. The Ti2p peak for the parent MOF appears at binding energy (B.E.) values of 488.9 and 464.6 eV (Figure 5a), which confirms the presence of Ti^4+^ for the titanium–oxo cluster, as demonstrated in previous studies [36]. After the metal exchange, both peaks are observed to shift towards lower binding energy, indicating that the presence of the new metal in the MOF structure increases the electron density of Ti [43]. However, no significant shift of the peaks is observed for Zr–NMIL–125, which may be attributed to the similar electronegativity of Zr and Ti [47,48]. On the other hand, the incorporation of Ce evidences a higher electron transfer, resulting in a higher electron density, and therefore displaces the peaks towards lower binding energies. After impregnation with Cu, all materials show similar behavior, i.e., a shift of Ti2p peaks towards lower binding energies, suggesting that the presence of copper consistently increases the electron density of titanium, regardless of the second metal incorporated in the MOF. Furthermore, the coordination of the incorporated metal ions (Zr, Ce and Cu) is confirmed by analysis of the N1s region (Figure S1b).

The N1s spectrum (Figure S1b) of the parent MOF was deconvoluted into three main peaks at 399.1, 401 and 402.7 eV and were assigned to the amino group (–NH_2_) and positively charged N species (–N=^+^, –NH^+^) [49,50]. In the case of the metal-exchanged catalysts, an interaction of the metal ions Zr^4+^, Ce^4+^ and Cu^2+^ with the amino groups of the precursor MOF is observed. In particular, the peak at 401 eV increases in intensity in these materials, which is associated with the formation of positively charged species (${–NH}_{2}^{+}$) [51], attributed to the donation of unshared electron pairs from the–NH_2_ group to the metal ions. For Ce-MIL-125(Cu), the peak located at 399.4 eV shifts toward lower binding energies, suggesting a higher electron density on nitrogen, probably due to a charge redistribution induced by the presence of the metal ions. This redistribution indicates a secondary interaction between the metals and the amino group of the MOF, which favors the partial transformation of the –NH_2_ into its quaternary form (${–NH}_{2}^{+}$), without implying a direct coordination with the incorporated metals. This effect is more pronounced in the case of Ce-MIL-125(Cu), which could be attributed to the participation of the Ce d-orbitals in the electronic redistribution and its larger atomic volume (20.7 cm^3^/g) compared to Zr (14 cm^3^/g) and Ti (10.5 cm^3^/g, which facilitates partial charge transfer within the MOF lattice and enhances the interaction with the amino groups.

Figure 5c shows the deconvolution of the Ce3d orbital spectra, indicating the coexistence of Ce^3+^ (E.B. of 880.1 eV, 885.3 eV, 899.8 eV and 904.1 eV) and Ce^4+^ (B.E. of 882 eV, 886.8 eV, 897.1 eV, 901.6 eV, 906.8 eV and 916.8 eV) dual oxidation states [47,52,53]. Likewise, the presence of Ce^3+^ could be attributed to the treatment of the material to carry out the XPS characterization since the equipment uses an ultra-high vacuum, which can favor the reduction of Ce^+4^ [52].

The spectrum of Zr3d (Figure 5d) showed two peaks at B.E. of 182.6 eV (Zr3d_5/2_) and 185.1 eV (Zr3d_3/2_) and the Zr–NMIL–125 presents a peak at 182.6 eV (Zr3d5/2) corresponding to the Zr^4+^ oxidation state [6,54,55]. Once the copper nanoparticles are incorporated the Zr3d peaks shift slightly to lower binding energies (182.3 eV), suggesting an increase in the electron density of Zr and in turn higher interaction between Cu and the SBUs of Zr–NMIL–125 [6,56]. In contrast, the binding energies of Cu2p (Figure 5b) in Zr–NMIL–125(Cu) shifted to higher binding energies, which suggests that Cu is coordinated with the Zr nodes. The Cu2p spectrum has two main peaks at B.E. of 932.5 eV (Cu^+^) and 934.8 eV (Cu^2+^) together with their satellite peaks [57,58]. These results clearly show the strong interaction between the Cu particles and the zirconium incorporated in the structure and as a consequence the electronic properties of the copper change significantly.

**Figure 6:**
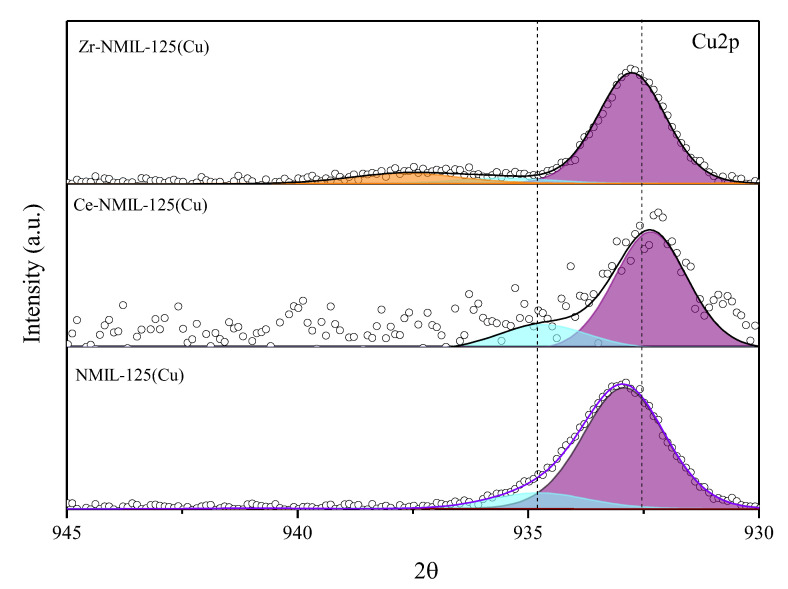
XPS spectra of Cu2p for the catalysts after reduction treatment.

Please note that a reduction treatment was carried out before the catalytic reaction (see Materials and Methods section). For that reason, we also analyzed the state of the catalyst elements after the reduction treatment, paying special attention to the state of Cu. The introduced Cu nanoparticles are expected to activate H_2_ dissociation and provide the catalyst the required activity, for which Cu^0^ is required. XPS results after the reduction (Figure 6) confirmed the oxidized Cu was transformed into metallic Cu species, as evidenced by the presence of a peak at 932.5 eV [31,59]. Likewise, the absence of satellite peaks, present on the catalysts before reduction (Figure 5), supports the conclusion that there are no Cu^n+^ species on the surface. As for Zr and Ce, no change in their oxidation states was observed after the reduction treatment (Figure S2).

### 2.2. CO_2_ Hydrogenation to Methanol Reaction

**Figure 7:**
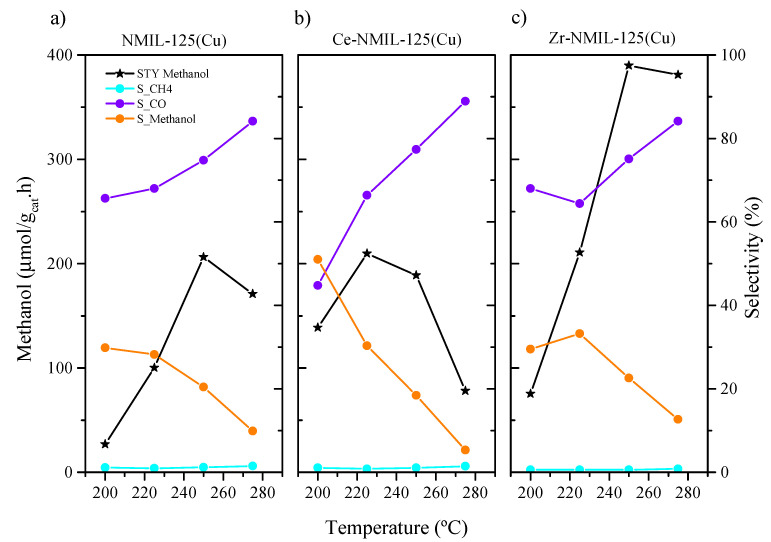
Influence of temperature on Space Time Yield (STY) and product selectivity for (**a**) NMIL–125(Cu), (**b**) Ce– NMIL–125(Cu) and (**c**) Zr–NMIL–125(Cu), in the following conditions: 30 bar, H_2_/CO_2_ ratio: 3/1 and a space time of 15 g h mol^−1^.

The synthesized catalysts were tested in the CO_2_ hydrogenation to methanol reaction in different experimental conditions. The CO_2_ conversions obtained were up to ca. 5% at a pressure of 30 bar, with the main reaction by-products being CO and methane. The unmodified NMIL–125 did not show any catalytic activity for the proposed reaction conditions (results are not included as they are not significant). Considering the results obtained with all the catalysts tested, only the results of the catalysts containing Cu in their composition, i.e., NMIL–125(Cu), Ce–NMIL–125(Cu) and Zr–NMIL–125(Cu), are presented below. Note that the presence of Cu was found to be required to convert CO_2_ (Figure S3). A series of experiments were conducted to evaluate the activity of NMIL–125(Cu), Ce–NMIL–125(Cu) and Zr–NMIL–125(Cu) and the effect of temperature during the hydrogenation of CO_2_ to methanol. These experiments were performed under a pressure of 30 bar, with an H_2_/CO_2_ ratio of 3/1 and a space time of 15 g h mol^−1^, across various temperatures: 200, 225, 250 and 275 °C. Figure 7 shows the results of this study. The CO_2_ conversion was as follows: 2.6%, 2.2% and 4.5% for NMIL–125(Cu), Ce–NMIL–125(Cu) and Zr–NMIL–125(Cu) catalyst, respectively (in Table S2 the values of CO_2_ conversion and selectivity to the products of the catalysts are presented). As for the methanol selectivity, it is evidenced that for both NMIL–125(Cu) and Ce–NMIL–125(Cu) catalysts their maxima are reached at a temperature of 200 °C and, as the reaction temperature increases, the methanol selectivity decreases, with that of CO increasing, which is due to the RWGS reaction being favored at a high temperature [58].

Comparing Space Time Yields (STYs) of methanol for each catalyst and knowing that they have similar amount of copper (between 27 and 30% atomic), it seems clear the improvement of the catalytic activity occurs when modifying the parent MOF with Ce or Zr. However, the methanol productivity tends to decrease using the Ce–NMIL–125(Cu) catalyst at temperatures above 225 °C, while the opposite occurs with the Zr–NMIL–125(Cu) catalyst. This latter presents higher methanol productivity values, reaching its maximum at 250 °C (380.8 µmol_methanol_/g_cat_·h). The results obtained with these catalysts are within the range obtained by other researchers with MOF-based catalysts composed of Zr and Cu. Kobayashi et al. [56] obtained a methanol STY value of 381.5 µmol_methanol_/g_cat_·h for the Cu/Zr–UiO–66–COOH catalyst. In another study, Stawowy et al. [34] reported similar results, obtaining an STY of 275 for catalysts based on UiO–66, with ion exchange (Zr for Ce) and Cu impregnation. These results align with ours and suggest that an improvement of the catalytic performance may be expected because of the enhanced interaction between the Cu particles and the Zr-exchanged MOF, which tentatively alters the electronic properties of the Cu particles. This finding also agrees with the work of Veliosoju et al. [31], who demonstrated that effective MOF-based catalysts for this reaction require a strong interaction between the MOF structure and the Cu particles, as shown in their study on Cu supported on ZIF–8. Similarly, Lecher’s group [46] published findings on the Zr-based MOF UiO–66, showing that the Cu–Zr interaction enhances the catalytic properties. The very low selectivity to methane in all cases is also worth mentioning, being almost null with the Zr–NMIL–125(Cu) catalyst, which is a promising result as methane is the least desired product in this reaction.

**Figure 8:**
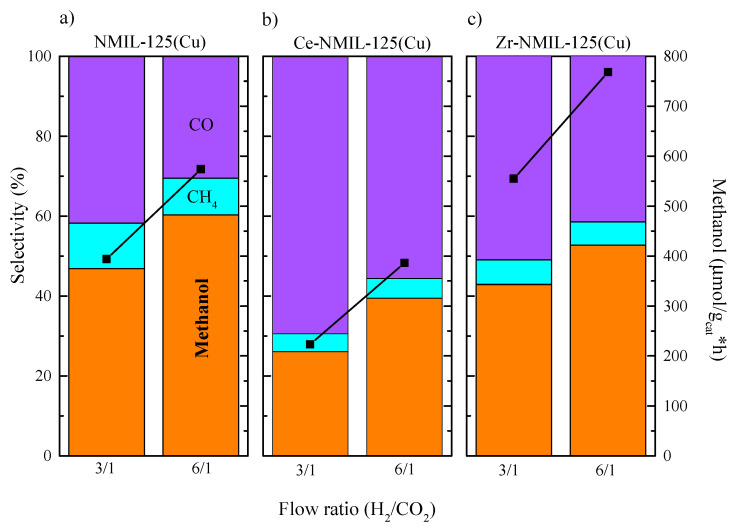
Influence of gas flow ratio (H_2_/CO_2_) on STY and product selectivity of (**a**) NMIL–125(Cu), (**b**) Ce– NMIL–125(Cu) and (**c**) Zr–NMIL–125(Cu), in the following conditions: 40 bar, H_2_/CO_2_ ratio: 3/1 and 6/1. The black line with squares refers to methanol (µmol_methanol_/g_cat_·h).

Setting the optimal temperature at 250 °C, we studied the influence of the total pressure (30 and 40 bar) and the H_2_/CO_2_ partial pressure (3/1 and 6/1 ratios) on the catalytic activity (Figure 8). A similar behavior is observed for the three catalysts. Contrary to what was expected, no significant changes in the conversion or product distribution were observed at 30 bar, which may be caused by the low conversion achieved in the used conditions. The catalysts containing Ce or Zr show the lowest selectivity towards methane (see Table S3).

At 40 bar pressure, the CO_2_ conversion, methanol selectivity and space time yields increase with the partial pressure of H_2_ in all cases. This was expected regarding the thermodynamics of the target reaction (Equation (1)), where an increase in the total pressure and an excess of H_2_ will shift the equilibrium reaction to the product side. The increases in methane selectivity in some cases may also be explained by the same reasoning. Otherwise, reverse water–gas shift should not be affected by the total pressure, explaining the better results achieved at 40 bar. Note that 40 bar was also the limitation of the experimental setup, for which higher pressure values were not tested. Again, Zr–NMIL–125(Cu) catalyst showed the highest activity for methanol production, with space time yields up to 768 µmol_methanol_/g_cat_·h. Even though methanol selectivity was slightly lower for the Zr-containing MOF than that of NMIL–125(Cu) catalyst (52.8% vs. 60.4%), Zr–NMIL–125(Cu) catalyst inhibited methane selectivity much more. This explains the overall better results, i.e., higher methanol STY and low methane selectivity. Likewise, Ce–NMIL–125(Cu) is the catalyst with a lower methanol STY, and it inhibits methane formation more than NMIL–125(Cu). In all cases, the increase in pressure plays an important role in the increase in the methanol STY, with 40 bar pressure and 6/1 flow rate being the conditions that maximized methanol productivity for the three catalysts, 348.1, 386.1 and 768.1 µmol_methano_l/g_cat_·h, for NMIL–125(Cu), Ce–NMIL–125(Cu) and Zr–NMIL–125(Cu), respectively.

At this stage, we can conclude that a significant influence of both partial metal exchange (Zr or Ce) and the incorporation of Cu nanoparticles has been found. Cu has been demonstrated to be required in order to activate H_2_ splitting. However, our results indicate that incorporating only Cu into the starting MOF does not enhance the catalytic activity as much as the combined incorporation of Zr + Cu or Ce + Cu does. Particularly, a better synergistic effect has been found between Zr–Cu, with Zr–NMIL–125(Cu) outperforming its Ce–NMIL–125(Cu) counterpart. This may be caused by an enhanced metal–support interaction, in this case Cu nanoparticles–NMIL–125 frameworks.

### 2.3. Characterization After Catalytic Tests

To evaluate the integrity of the catalysts after their use, a post-reaction characterization was performed. X-ray diffraction (XRD) analyses, presented in Figure S4a, show that the three catalysts preserved their crystallinity after 24 h under the stipulated reaction conditions. In contrast, the N_2_ adsorption isotherms, illustrated in Figure S4b, reveal a significant reduction in the N_2_ adsorption capacity of the three catalysts, which implies a corresponding decrease in their specific surface area. This has been systematically reported in nanoparticle-containing MOFs, but most surprisingly, as in our case, neither the surface area nor the linker functionalization limits the CO_2_ hydrogenation activity, which remains constant [29]. Moreover, no significant changes in the oxidation state were detected by XPS (Figure S5) or in the morphology observed by SEM or TEM (Figure S6). In fact, the disk-like structure of the parent MOF remains unchanged after the reaction. These findings corroborate the stability of the NMIL–125 metal–organic structure, which not only resisted the proposed post-synthetic modifications but also withstood the reaction conditions (temperature and pressure) without losing significant efficiency. This demonstrates the viability of this catalytic system for methanol production from CO_2_ hydrogenation, maintaining its activity and stability.

## 3. Materials and Methods

### 3.1. Materials

Titanium (IV) isopropoxide (Ti[OCH(CH_3_)_2_]_4_), aminoterephthalic acid, ammonium cerium (IV) nitrate, zirconium (IV) oxynitrate hydrate, copper (II) acetate, N–dimethylformamide (DMF, 99.8%) and methanol (99%) were used. All reagents were purchased from Sigma–Aldrich Co. (St. Louis, MI, USA).

### 3.2. Synthesis of the Materials

Pristine NH_2_-MIL–125 MOF was synthesized using a solvothermal method. First, 6 mmol of the ligand (aminoterephthalic acid) was dissolved in a 1:1 mixture by volume of DMF and methanol; once dissolved, 3 mmol of the metallic precursor (titanium isopropoxide) was added. The obtained solution was transferred to a stainless steel jacketed Teflon autoclave and kept in an oven at 150 °C for 4 h. The resulting yellow powder was separated by centrifugation, washed twice with DMF to remove the unreacted ligand and twice with methanol to remove the remaining DMF and finally the material was dried overnight at 150 °C under vacuum. The nomenclature used for NH_2_–MIL–125 MOF is that of the pristine MOF, i.e., NMIL–125.

NMIL–125 bimetallic based MOFs: The materials based on the NMIL–125 structure with the incorporation of a new metal M (M: Zr, Ce, Cu) were synthesized in a glass bottle with a cap, where the metal salt (ammonium cerium (IV) nitrate, zirconium (IV) oxynitrate hydrate or copper (II) acetate, as appropriate) was dissolved in a 1:1 mixture (50 mL) of DMF and methanol. Once the salt was dissolved, 500 mg of NMIL–125 was added to the solution, which was magnetically stirred for 30 min at room temperature. The mixture was kept in an oven at 80 °C for 72 h. The powder obtained was recovered by filtration and washed with methanol for 6 h in a Soxhlet extraction system. The nomenclature for the metal-modified MOF is M–NMIL–125 (M: Zr or Ce).

Impregnation of bimetallic MOFs with copper: After obtaining the bimetallic MOFs, copper impregnation was performed using the same procedure for all bimetallic MOFs. In a glass bottle with a cap containing 50 mL of a 1:1 mixture of DMF and methanol, copper (II) acetate was dissolved. Once the salt was dissolved, 500 mg of bimetallic MOF was added to the mixture and kept in an oven at 70 °C for 72 h. The resulting powder was recovered by filtration and washed with methanol for 6 h in a Soxhlet extraction system. The nomenclature used for the impregnated MOF is M–NMIL–125(Cu).

### 3.3. Characterization

The crystalline structure of the materials was determined by powder X-ray diffraction (PXRD) recorded on Panalytical Empyrean Multifunctional X-ray diffraction analysis equipment, which in its basic configuration has a goniometer with an X-ray tube with a Cu Kα cathode and Ni filter. It was operated in an angular scanning range of 5° to 35° at an angular velocity of 1°/min and at room temperature.

The textural parameters of the materials were determined by N_2_ adsorption–desorption. The samples were subjected to a degassing treatment at 250 °C in vacuum for 8 h before adsorption measurements. Nitrogen adsorption–desorption isotherms were collected at –196 °C in a Quadrawin device (Quantachrome). The surface area was determined from the N_2_ adsorption branch. In all cases, the number of points used to apply the BET equation was greater than 5, and the value of C was always positive [60,61]. The micropore volume (V_micro_) was estimated by the Dubinin–Raduskevich method, the mesopore volume (V_meso_) was calculated from the difference between the total volume (liquid volume) of N_2_ adsorbed at a relative pressure of 0.90 and the non-local density functional theory (NLDFT) has been used for the pore size distribution.

The content of the incorporated metals (Zr, Ce and Cu) was determined by inductively coupled plasma–optical emission spectroscopy (ICP–OES) (Agilent Technologies, Melbourne, Australia).

The morphology of the samples was analyzed by field emission scanning electron microscopy with X-ray microanalysis (FESEM–EDS) (ZEISS–Merlin VP Compact, BRUKER Quantax 400, Bruker, MA, USA) in the backscattered electron (BSE) and secondary electron (SE) modes. Also, some of the samples have been analyzed by transmission electron microscopy (TEM) (JEOL JEM–1400 Plus, Jeol, Tokio, Japan). 

The X-ray photoelectron spectroscopy (XPS) study was performed in a Thermo–Scientific Kα spectrometer (Thermo Fisher Scientific, Waltham, MA, USA). Strict peak deconvolution was carried out, and the peak areas were estimated by calculating the integral of each peak. The Shirley background was subtracted, and the experimental peak was fitted to a Lorentzian/Gaussian combination with a 30/70 ratio.

Thermogravimetric mass spectroscopy (TG–MS) curves were recorded on a TGA/STA 449 F5 Jupiter coupled to an Aeolos QMS 403 Quadro quadrupole mass spectrometer, both from NETZSCH (Selb, Germany). The samples were exposed to a temperature range of 25–1000 °C increasing by 10 °C min^−1^, while the air flow rate was kept constant (50 mL–min^−1^).

### 3.4. Catalytic Test

Catalytic tests were conducted using a fixed-bed stainless steel reactor within a Microactivity Effi setup (PID Eng & Tech, Madrid, Spain), employing 200 mg of catalyst. The reactor was heated by a ceramic oven and temperature was measured inside the bed using a K-type thermocouple. The pressure was regulated with a back pressure regulator. The materials were pelletized and sieved to a particle size of 250–425 μm. These pellets were then arranged on a bed of silica balls to ensure the placement of the catalyst within the isothermal zone of the reactor. Before the reactions, catalysts underwent a pre-treatment process in an atmosphere of 10% H_2_ and 90% N_2_ at 250 °C for 4 h. Catalyst activity was evaluated using several experimental batches. In one batch, CO_2_ and H_2_ flow rates and pressure were held constant, while temperature was varied. In another batch, the temperature (250 °C) was kept constant while varying the pressure (30 bar and 40 bar) as well as the flow rate of the gases (H_2_/CO_2_: 3/1 and 6/1). The reaction products were analyzed in-line using a gas chromatograph (Scion 456–GC, Scion Instruments, Livingston, Scotland, UK) equipped with three detectors: one TCD and two FIDs. The indexes for evaluating performance included conversion (X_CO2_, %), selectivity (Si, %) and yield (Yi, %), defined as follows:

(5) $$X_{{\mathrm{CO}}_{2}}\left(\%\right)=\frac{\sum n_{i}F_{i}}{F_{{\mathrm{CO}}_{2}}+\sum n_{i}F_{i}}\;\times \;100$$

(6) $$S_{i}=\frac{n_{i}F_{i}}{\sum n_{i}F_{i}}\;\times 100$$

(7) $$Y_{i}=X_{CO_{2}}\;\times S_{i}$$

where *n_i_* is the number of carbons of each product *i* and *F_CO2_* and *F_i_* are the molar flow rates of CO_2_ and each product *i* at the outlet of the reactor.

## 4. Conclusions

Catalysts based on the NMIL–125 metal–organic framework were prepared by an ion exchange post-synthetic procedure, achieving the incorporation of a second metal into the structure, resulting in an optimal support on which copper nanoparticles were deposited. The successful incorporation of M^+^ cations (Ce^4+^, Zr^4+^ and Cu^2+^) was confirmed by extensive characterization, including techniques such as XRD, N_2_ adsorption, TGA and XPS. Crystalline and microporous materials with a slightly smaller specific surface area than the original MOF were obtained, confirming the integration of M^+^ cations into the MOF structure. XPS spectra confirmed the presence of Zr, Ce and Cu species in the materials. However, the Cu was demonstrated to be almost fully reduced in the pre-treatment. Catalytic tests under the selected test conditions showed that the Zr–NMIL–125(Cu) catalyst presented the highest CO_2_ conversion (max. of 4.6%), STY of methanol (max. of 768 mol/g_cat_.h) and selectivity towards methanol, besides almost inhibiting methane production. The synergetic interaction between the Zr-containing support (MOF) and the Cu nanoparticles was the key factor for the enhanced catalytic activity. The results of this study demonstrate that the interaction between the MOF and Cu nanoparticles is crucial for enhancing catalytic activity in the CO_2_ hydrogenation to methanol.

## Figures and Tables

**Table 1:** Inductively coupled plasma emission spectroscopy (ICP–OES) analysis of the synthesized catalysts.

	at%				Ratio	Formula Units		
Sample	Ti	Zr	Ce	Cu	Ti/M	Ti	Zr	Ce
NMIL–125	100	––	––	––	––	8	––	––
Ce–NMIL–125	85.7	––	14.3	––	6.0	6.86	––	1.14
Zr–NMIL–125	81.0	19.0	––	––	4.3	6.48	1.52	––
NMIL–125(Cu)	72	––	––	28.0	––	––	––	––
Ce–NMIL–125(Cu)	60.3	––	10.2	29.5	5.9	––	––	––
Zr–NMIL–125(Cu)	60.4	12.2	––	27.4	4.9	––	––	––

**Table 2:** Textural properties of as-synthesized catalysts.

Sample	Specific Surface Area BET (m^2^/g)	Vmicro (cm^3^/g)	Vmeso (cm^3^/g)	Average Pore Size (nm)
NMIL–125	1452	0.57	0.11	0.65
Ce–NMIL–125	914	0.36	0.11	0.65
Zr–NMIL–125	847	0.34	0.17	0.66
NMIL–125(Cu)	1122	0.44	0.19	0.64
Ce–NMIL–125(Cu)	805	0.32	0.21	0.66
Zr–NMIL–125(Cu)	581	0.23	0.18	0.65

## Data Availability

All data generated or analyzed during this study are included in the published article and its Supplementary Materials.

## Supplementary Materials

The following supporting information can be downloaded at: www.mdpi.com/article/10.3390/molecules30071458/s1, Figure S1: CO_2_ adsorption and XPS spectra of fresh catalysts: (a) C1s. (b) N1s and (c) O1s. Figure S2: XPS spectra of catalysts after reduction pre-treatment: (a) Ce3d, (b) Zr3d. Figure S3. Influence of temperature on CO_2_ conversion of catalyst without Cu. Figure S4: (a) Post-reaction XRD patterns of the as-synthesized NMIL–125 and the mixed metal NMIL–125-based catalyst, (b) N_2_ adsorption isotherms of catalysts after reaction. Figure S5: XPS spectra of catalysts post-reaction: (a) Tip2, (b) Cu2p, (c) Ce3d, (d) Zr3d. Figure S6: FESEM images: (a) NMIL–125(Cu), (b) Ce-NMIL–125(Cu), (c) Zr-NMIL–125(Cu). And TEM images: (d) NMIL–125(Cu), (e) Ce-NMIL–125(Cu), (f) Zr-NMIL–125(Cu). Table S1: Surface chemical composition of the fresh catalysts obtained by XPS. Table S2: CO_2_ conversion, product selectivity and STY at different temperatures. Table S3: CO_2_ conversion, product selectivity and STY at different pressures and gas flow ratios. Table S4: Surface chemical composition of the catalysts post-reaction obtained by XPS.
