# Supplementary material for: Tailoring Catalysts for CO2 Hydrogenation: Synthesis and Characterization of NH2–MIL–125 Frameworks
Source: Molecules. 2025 Mar 25;30(7):1458. doi: 10.3390/molecules30071458 (PMC11990164; doi:10.3390/molecules30071458)
Supplement: Supplementary file 1 [file molecules-30-01458-s001.zip › molecules-3521505-supplementary.pdf]

Fresh Catalysts Characterization

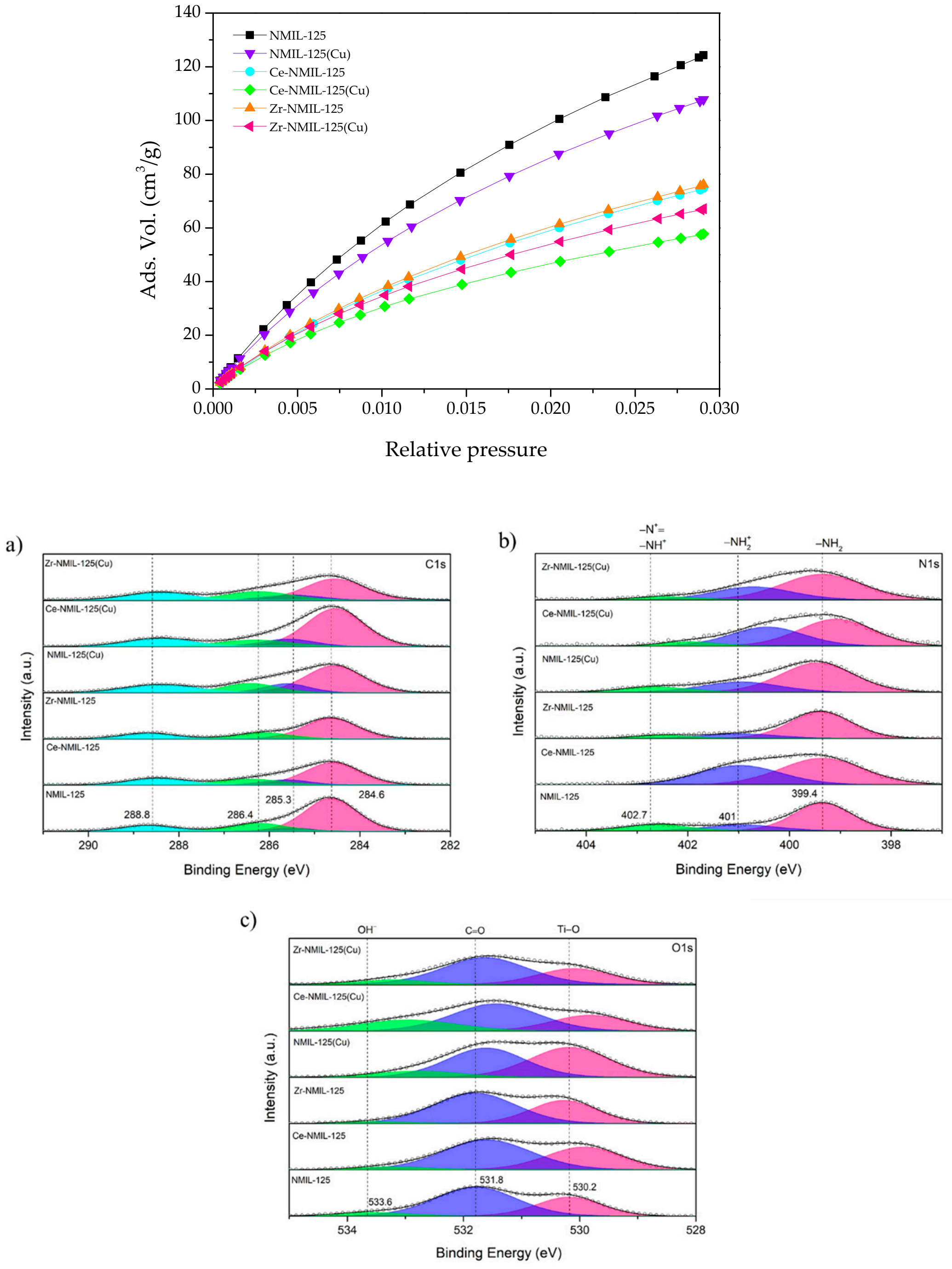

Figure S1. CO^{2} adsorption and XPS spectra of fresh catalysts: (**a**) C1s. (**b**) N1s and (**c**) O1s.

Table S1. Surface chemical composition of the fresh catalysts obtained by XPS.

	Element (wt%)						
**Catalyst**	**C**	**O**	**N**	**Ti**	**Ce**	**Zr**	**Cu**
NMIL–125	45.18	28.25	4.19	22.39	—	—	—
Ce–NMIL–125	31.59	29.74	4.86	17.62	16.18	—	—
Zr–NMIL–125	28.25	32.8	3.78	15.57	—	19.59	—
NMIL–125(Cu)	29.41	31.91	4.64	22.98	—	—	11.06
Ce–NMIL–125(Cu)	37.95	25.91	2.54	8.83	12.92	—	11.84
Zr–NMIL–125(Cu)	20.2	28.74	4.69	16.23	—	10.61	19.52

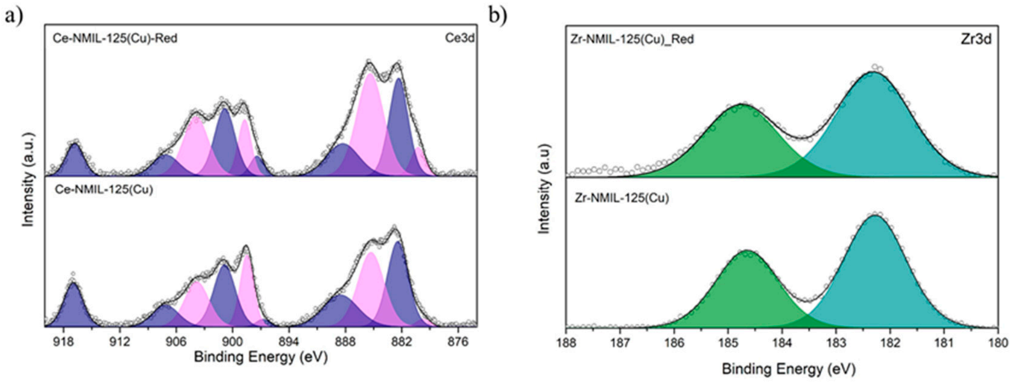

Figure S2. XPS spectra of catalysts after reduction pretreatment: (**a**) Ce3d, (**b**) Zr3d.

Table S2. CO_2 conversion, product selectivity ans STY at differents temperatures.

	**Temp. (ºC)**	**X_CO$_2$**	**S_CO**	**S_CH$_4$**	**S_Methanol**	**Methanol (µmol/g$_{cat}$*h)**
NMIL–125(Cu)	200	0.1	65.7	4.5	29.9	27.0
	225	0.5	68.0	3.8	28.2	100.3
	250	1.5	74.8	4.8	20.4	206.2
	275	2.6	84.2	5.9	9.9	170.9
Ce–NMIL–125(Cu)	200	0.4	44.8	4.2	51.0	138.8
	225	1.0	66.4	3.3	30.4	210.7
	250	1.5	77.4	4.2	18.4	188.7
	275	2.2	89.0	5.7	5.3	77.9
Zr–NMIL–125(Cu)	200	0.4	68.0	2.5	29.5	73.6
	225	1.0	64.4	2.4	33.2	210.5
	250	2.6	75.1	2.3	22.6	389.7
	275	4.5	84.1	3.2	12.7	380.9

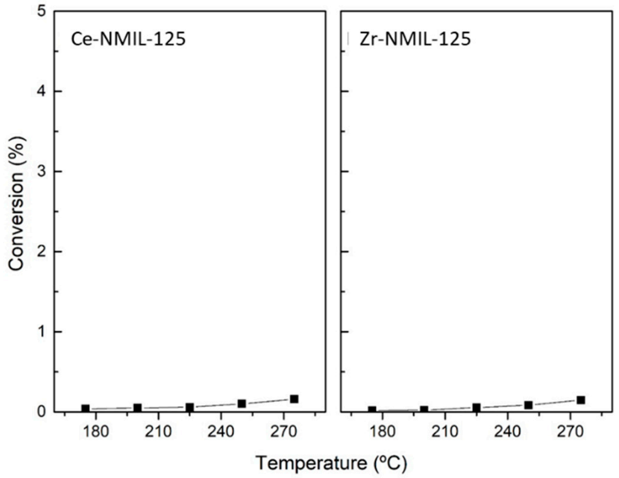

Figure S3. Influence of temperature on CO_2 conversion of catalyst without Cu.

Table S3. CO_2 conversion, product selectivity and STY at differents pressure and gass flow ratio.

	Pressure (bar)	Flow ratio H_2/CO_2	X_CO_2	S_CO	S_CH_4	S_Methanol	Methanol (µmol/g_{cat}*h)
NMIL–125(Cu)	30	3/1	1.3	51.1	8.9	39.9	348.1
	30	6/1	1.2	51.7	10.7	37.6	290.7
	40	3/1	1.3	41.8	11.4	46.9	394.0
	40	6/1	1.4	30.5	9.2	60.4	573.8
Ce–NMIL–125(Cu)	30	3/1	1.1	74.9	4.2	20.9	159.4
	30	6/1	1.2	79.6	2.5	17.9	137.4
	40	3/1	1.3	69.5	4.5	26.1	223.1
	40	6/1	1.5	55.6	5.0	39.4	386.1
Zr–NMIL–125(Cu)	30	3/1	1.8	53.4	5.9	40.8	488.0
	30	6/1	1.7	62.6	6.2	31.2	344.1
	40	3/1	1.9	51.0	6.1	42.9	555.0
	40	6/1	2.2	41.5	5.8	52.8	768.1

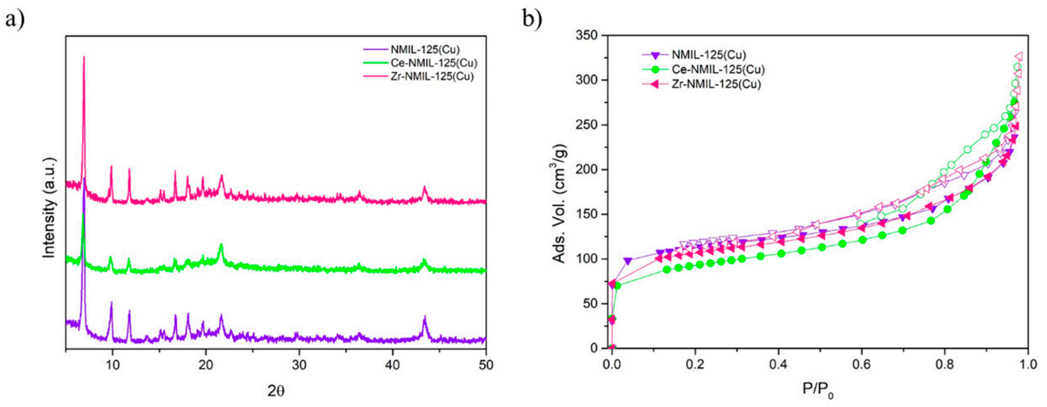

Figure S4. (**a**) Post-reaction XRD patterns of the as-synthesized NMIL-125 and the mixed metal NMIL-125 based catalyst. (**b**) N_2 adsorption isotherms catalysts after reaction.

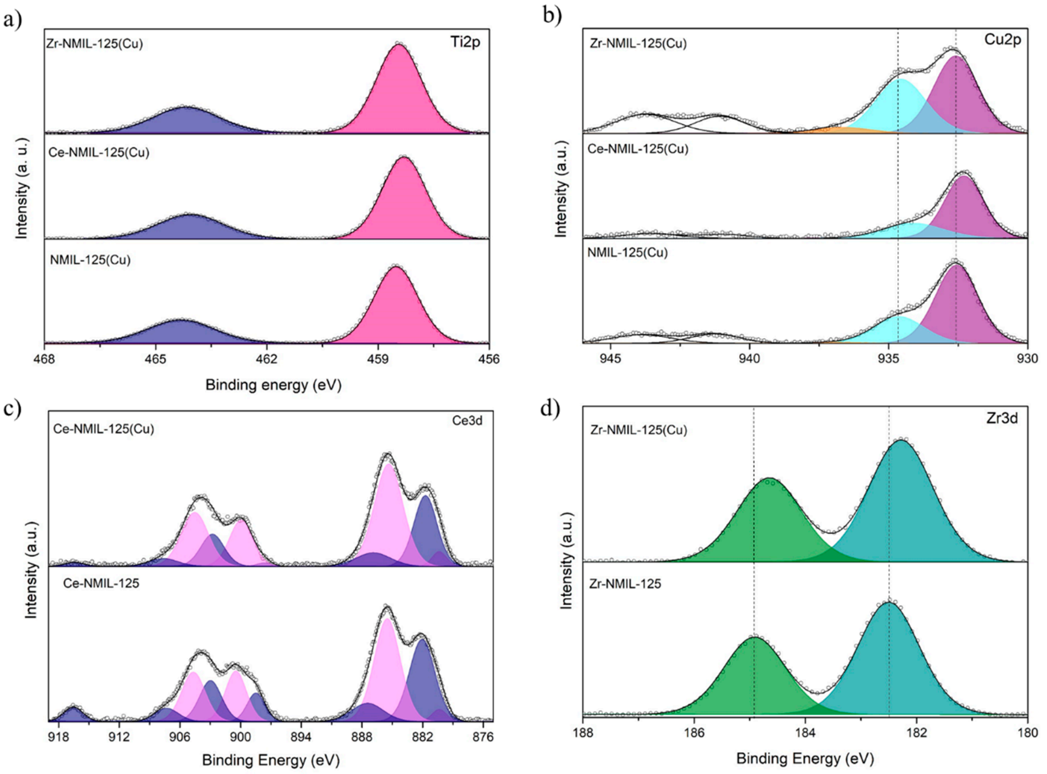

Figure S5. XPS spectra of catalysts post-reaction: (**a**) Tip2, (**b**) Cu2p, (**c**) Ce3d, (**d**) Zr3d.

Table S4. Surface chemical composition of the catalysts post reaction obtained by XPS.

	Element (wt%)						
**Catalyst**	**C**	**O**	**N**	**Ti**	**Ce**	**Zr**	**Cu**
NMIL–125	40.46	33	4.98	21.39	—	—	—
Ce–NMIL–125	29.01	28.09	3.05	14.09	24.95	—	—
Zr–NMIL–125	33.67	31.17	3.99	15.57	—	15.6	—
NMIL–125(Cu)	29.65	33.28	3	25.42	—	—	8.65
Ce–NMIL–125(Cu)	25.86	29.64	2	19.77	17.96	—	4.78
Zr–NMIL–125(Cu)	23.58	30.23	1.96	18.37	—	13.35	12.51

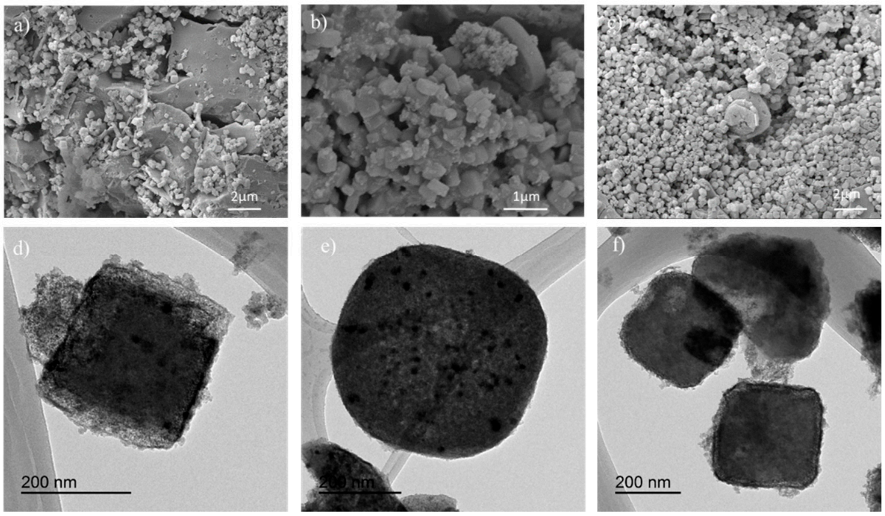

Figure S6. FESEM images: (**a**) NMIL-125(Cu), (**b**) Ce-NMIL-125(Cu), (**c**) Zr-NMIL-125(Cu). And TEM images: (**d**) NMIL-125(Cu), (**e**) Ce-NMIL-125(Cu), (**f**) Zr-NMIL-125(Cu).
